# Global research output on HIV/AIDS–related medication adherence from 1980 to 2017

**DOI:** 10.1186/s12913-018-3568-x

**Published:** 2018-10-10

**Authors:** Waleed M. Sweileh

**Affiliations:** Department of Physiology, Pharmacology/Toxicology, Division of Biomedical Sciences, College of Medicine and Health Sciences, An-Najah National University, Nablus, Palestine

**Keywords:** Medication adherence, HIV, AIDS, Bibliometric analysis, VOSviewer, ArcGIS 10.1

## Abstract

**Background:**

“Human Immunodeficiency Virus (HIV)” and Acquired Immunodeficiency Syndrome (AIDS) are global health burden. Medication adherence in people living with HIV (PLWH) is a key element in reducing morbidity and mortality. Quantitative and qualitative assessment of research activity helps identify research gaps as well as efforts implemented to improve adherence behaviors in PLWH. The aim of the current study was to assess and analyze literature on HIV/AIDS–related medication adherence using bibliometric methods.

**Methods:**

SciVerse Scopus was used to accomplish the purpose of the current study. The study period included all times up to 2017. The analysis was restricted to documents published in academic journals.

**Results:**

Search strategy retrieved 3021 documents with an average of 32.5 citations per document, an *h*-index of 136, and an average of 4.4 authors per documents. The volume of literature on HIV/AIDS-related medication adherence constituted 1.3% of the overall HIV/AIDS literature. There was a significant (*p* < 0.01; *r* = 0.9) correlation between the growth of publications in AIDS-related stigma and medication adherence. The regions of America (567.9) had the highest research output per one million infected people (567.9) followed by the European region (314.3), Western Pacific Region (70.7), Eastern Mediterranean region (31.4), South East Asia (34.0), and Africa (19.3). Geographical distribution of publications showed an active contribution of certain countries in the Southern and Eastern region of Sub-Saharan Africa. *Harvard University* (8.4%; *n* = 254) was the most active institution. The top cited documents focused on the impact of adherence on disease outcome and the impact of text messages on improving medication adherence.

**Conclusion:**

Research on medication adherence in PLWH showed regional variations. International research collaboration with high burden regions such as Sub-Saharan Africa needs to be strengthened to achieve the global target of ending AIDS as a public health threat by 2030.

**Electronic supplementary material:**

The online version of this article (10.1186/s12913-018-3568-x) contains supplementary material, which is available to authorized users.

## Background

Human Immunodeficiency Virus (HIV) and its clinical manifestation of “Acquired Immunodeficiency Syndrome (AIDS)” are considered major global public health challenge [1, 2]. It is estimated that 36.7 million people worldwide are living with HIV and approximately one million died of HIV-related causes globally [3]. Currently, no cure is available for HIV infection. However, effective antiretroviral therapy (ART) is considered the optimum available therapy for people living with HIV (PLWH) and those at substantial risk [4]. The early use of combination ART, known as highly active antiretroviral treatment (HAART), has resulted in increased survival in PLWH [5]. In 2016, World Health Organization (WHO) released the guideline for treating and preventing HIV infection which included recommendations to provide lifelong ART to all categories of PLWH regardless of their CD4 cell count [6]. According to WHO fact sheet updated in November 2016, expanding ART therapy to all PLWH and expanding prevention choices can help minimize millions of deaths and new infections expected by the year 2030 [7].

Prevention and control of HIV infection largely depend on adherence to recommended treatment [8, 9]. Poor adherence, especially at levels less than 95%, adversely affects the HIV outcome [10–13]. Adherence can be problematic in low and middle-income countries because of inadequate healthcare services, limited health literacy, and lack of social support [14–17]. Even in developed countries, less than half of surveyed patients had 100% adherence to HIV medications [18]. Studies on adherence to recommended HIV therapy as well as barriers to 100% adherence are, therefore, vital in all world regions and countries. Actually, the importance of high-level adherence to HIV therapy had resulted in a vast number of publications that investigated factors associated with non-adherence and beneficial strategies that can be used to overcome such factors [19–21]. Interventional studies to improve medication adherence in PLWH included implementation of recent mobile technology which proved to be cost-effective compared with other interventional studies [22].

The need to assess research trends in medication adherence in PLWH is important for researchers, health policymakers, and clinical practitioners. Such importance is due to the crucial role of adherence in improving survival of infected people and minimizing the spread of infection given that medication adherence varies with different societies and different world regions [23, 24]. Assessing growth and research trends are also important for improving the volume and quality of research, identifying research gaps that future studies need to focus on, and identifying geographical spots with low research productivity relative to their HIV/AIDS national burden. To date, no studies had been published to summarize global research efforts, research trends, and geographical distribution of research output in medication adherence in general and in PLWH in particular. It should be emphasized here that several reports have been published about HIV/ AIDS research productivity in general [25–28], but none was published about research output on medication adherence in specific. Therefore, the aim of this study was to give a comprehensive analysis, both quantitative and qualitative, of scientific literature on medication adherence in PLWH.

## Methods

In this study, a bibliometric methodology was implemented. Bibliometric analysis is not the same as systematic reviews or scoping reviews. In bibliometric analysis, one single database is used to provide baseline information and to identify research gaps for future studies and funding purposes [29, 30]. SciVerse Scopus or Web of Knowledge is usually selected to carryout bibliometric analysis. No grey literature is included in bibliometric analysis. Therefore, bibliometric analysis is not comprehensive of all literature. In contrast, systematic reviews have a specific research question that needs to be answered using a limited number of publications. In systematic reviews, literature published in different databases, including grey literature, is retrieved and filtered based on a preset inclusion and exclusion criteria. This is the reason why in systematic reviews, but not in bibliometric analysis, the retrieved documents will include a large percentage of duplicate documents. In systematic reviews, researchers might follow up with the analysis and carry out meta-analysis, which is not the case in bibliometric analysis [31, 32]. For scoping reviews, the nature and extent of research evidence are analyzed [33, 34].

### Search strategy and keywords

SciVerse Scopus, an online database containing abstracts and citations of more than 23,000 journals in various fields [35], was used to achieve the purpose of this study. Furthermore, SciVerse Scopus was used since it was previously used in several bibliometric studies including those pertaining to HIV/AIDS [36–44]. The implemented search strategy was shown in the additional file (Additional file 1). Quotation marks and asterisks were used in the search strategy to enhance the accuracy and comprehensiveness of the results. The search strategy consisted of combined search queries of keywords related to HIV/AIDS and keywords related to medication adherence. Keywords used were partially obtained from previously published literature on AIDS-related medication adherence [21, 23, 45, 46]. An exclusion step was implemented to eliminate potential false positive documents. This study was limited to documents published in academic journals any time up to 2016.

### Bibliometric indicators and mapping

The quality of retrieved publications was assessed using Hirsh-index (*h*-index) [47]. Author keywords were also analyzed and visualized using VOSviewer software [48]. ArcGIS 10.1 software was used to map the geographic distribution of the retrieved literature [49]. The analysis also included distribution of research output based on world health organization (WHO) world regions [50]. For the calculation of the number of publications per one million PLWH, the WHO data on numbers of infected people in each region was used [51]. Prevalence data for each of the top ten counties were obtained from CIA World Factbook [52]. All data presented in this study were obtained by analysis of data retrieved on July 29, 2018.

## Results

### Types of retrieved documents

The search strategy retrieved 3021 journal documents. The same search strategy retrieved approximately 232,000 documents on HIV/AIDS. Therefore, the literature on medication adherence in PLWH constituted 1.3% of total literature on HIV/AIDS published during the same study period. Retrieved documents were mostly research articles (2520; 83.4%) and review articles (255; 8.4%). The types of documents encountered in the retrieved literature were shown in Table 1. Thirteen different languages were encountered in the abstracts of retrieved articles, mostly English (2857; 94.6%) and Spanish (99; 3.3%). In total, 642 (21.3%) documents were published in journals within the scope of psychology while 401 (13.3%) documents were published in journals within the scope of social sciences and humanities. The remaining documents (1972; 65.5%) were published in various journals within biomedical and health fields.

**Table 1:** Types of retrieved documents

Types of document	Number of publications *N* = 2031	%
Article	2520	83.4
Review	255	8.4
Letter	100	3.3
Conference Paper	61	2.0
Note	42	1.4
Editorial	27	0.9
Short Survey	15	0.5
Article in Press (undefined)	1	0.0

### Most frequent keywords

**Fig. 1:**
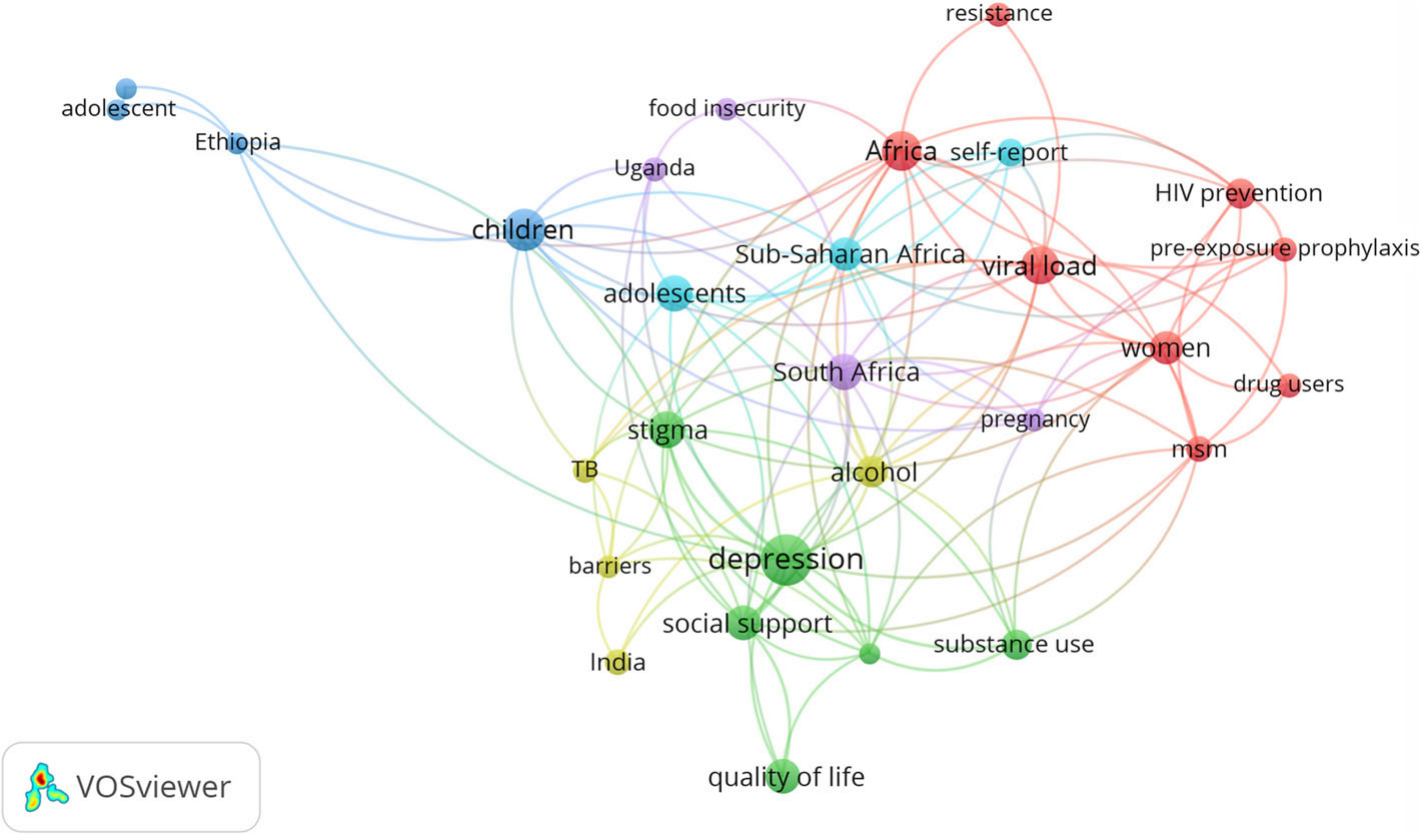
Network visualization map of author keywords with a minimum frequency of 10 times

Analysis of the most frequent keywords, excluding those pertaining to HIV/AIDS and adherence, were shown in Fig. 1. The most frequently encountered author keywords in retrieved the literature were depression/depressive symptoms, stigma, /substance use, Sub- Saharan Africa, children, viral load, poverty/food insecurity, quality of life, and several others.

### Growth of publications

**Fig. 2:**
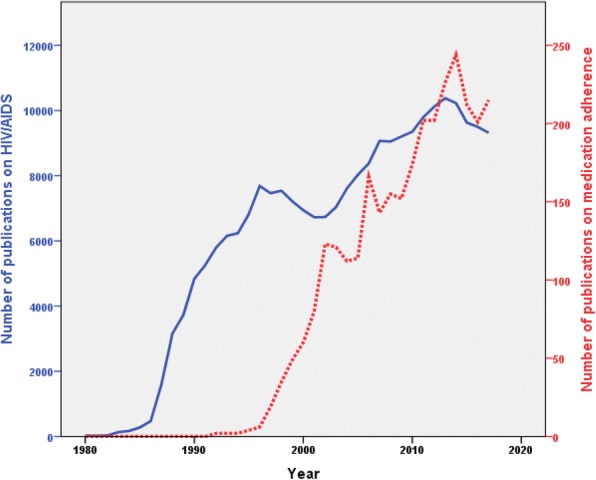
Growth of publications on HIV/AIDS – related medication adherence relative to the overall growth of publications on HIV/AIDS in general

Publications on HIV/AIDS-related medication adherence started as early as 1992. The number of publications increased with time and reached a maximum of approximately 200 documents per year in the last five years of the study period. Table 2 shows the annual number of publications on medication adherence and the annual number of publications on HIV/AIDS in general. The percentage of publications on medication adherence from the corresponding number of HIV/AIDS publications showed a linear increase with time. The growth of publications on medication adherence started one decade later than that on HIV/AIDS. The growth of publications on medication adherence was parallel to that of HIV/AIDS until early 2000 (Fig. 2). In the last decade of the study period, the growth of publications on medication adherence showed stronger upward increase than that of HIV/AIDS.

**Table 2:** Growth of publications on HIV/AIDS in general and publications on medication adherence in specific

Year	Number of publications on medication adherence	Number of overall publications on HIV/AIDS	% of publication on medication adherence from overall publications	Year	Number of publications on medication adherence	Number of overall publications on HIV/AIDS	% of publication on medication adherence from overall publications
1980	0	21	0	1999	49	7213	0.68
1981	0	20	0	2000	60	6941	0.86
1982	0	27	0	2001	80	6726	1.19
1983	0	131	0	2002	123	6732	1.83
1984	0	168	0	2003	121	7037	1.72
1985	0	275	0	2004	112	7611	1.47
1986	0	468	0	2005	114	8027	1.42
1987	0	1615	0	2006	166	8376	1.98
1988	0	3163	0	2007	143	9066	1.58
1989	0	3727	0	2008	155	9051	1.71
1990	0	4835	0	2009	152	9203	1.65
1991	0	5256	0	2010	174	9350	1.86
1992	2	5794	0.03	2011	202	9792	2.06
1993	2	6156	0.03	2012	202	10,118	2
1994	2	6237	0.03	2013	226	10,377	2.18
1995	4	6820	0.06	2014	244	10,230	2.39
1996	6	7684	0.08	2015	212	9628	2.2
1997	19	7465	0.25	2016	201	9498	2.12
1998	35	7539	0.46	2017	215	9316	2.31

### Growth of citations

**Fig. 3:**
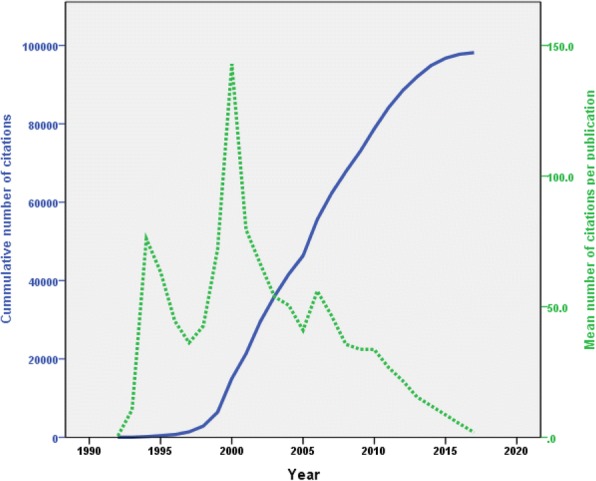
Annual and cumulative number of citations

Retrieved publications received 98,175 citations, an average of 32.5 citations per document, and an *h*-index of 136. Table 3 shows the mean number of citations per publication per year and the cumulative number of citations. The mean number of citations was highest for documents published in early 2000 shown as a peak in Fig. 3. The cumulative number of citations, on the other hand, showed a linear upward pattern.

**Table 3:** Mean number of citations per document per year and cumulative number of citations

Year	Number of publications on medication adherence	Number of citations	Mean number of citations per document	Cumulative number of citations	Year	Number of publications on medication adherence	Number of citations	Mean number of citations per document	Cumulative number of citations
1992	2	1	0.5	1	2005	114	4679	41.0	46,330
1993	2	21	10.5	22	2006	166	9293	56.0	55,623
1994	2	152	76.0	174	2007	143	6649	46.5	62,272
1995	4	253	63.3	427	2008	155	5525	35.6	67,797
1996	6	267	44.5	694	2009	152	5115	33.7	72,912
1997	19	688	36.2	1382	2010	174	5841	33.6	78,753
1998	35	1490	42.6	2872	2011	202	5391	26.7	84,144
1999	49	3523	71.9	6395	2012	202	4369	21.6	88,513
2000	60	8571	142.9	14,966	2013	226	3477	15.4	91,990
2001	80	6358	79.5	21,324	2014	244	2939	12.0	94,929
2002	123	8163	66.4	29,487	2015	212	1830	8.6	96,759
2003	121	6508	53.8	35,995	2016	201	1016	5.1	97,775
2004	112	5656	50.5	41,651	2017	215	400	1.9	98,175

### Geographical distribution of publications

**Fig. 4:**
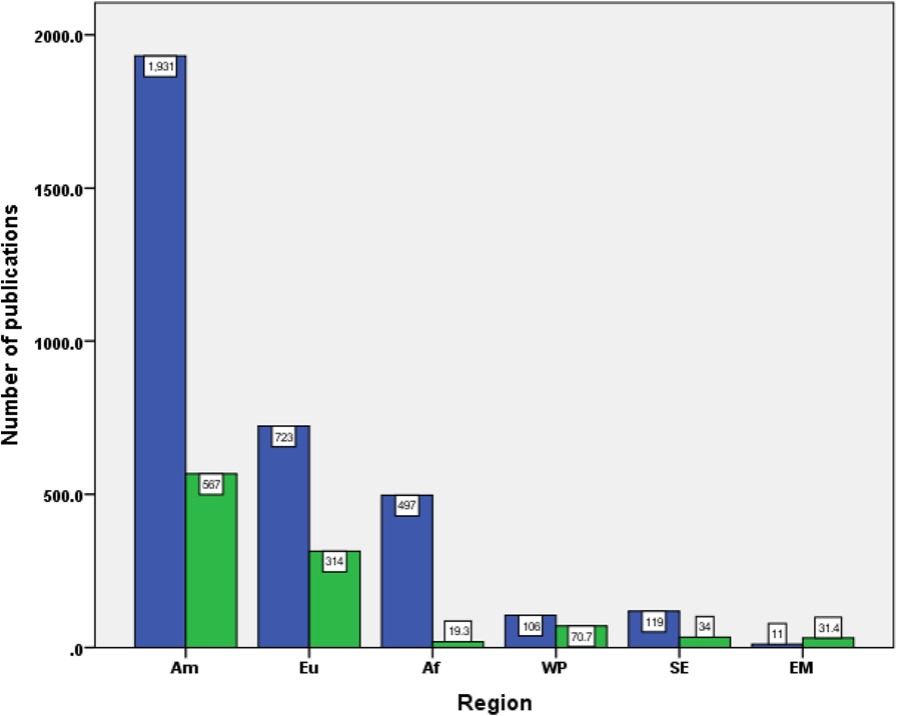
contribution of different WHO regions to retrieved literature adjusted by the number of HIV infected people. Am: Americas; Eu: Europe; Af: Africa; WP: Western Pacific; SE: South Eastern; EM: Eastern Mediterranean

**Fig. 5:**
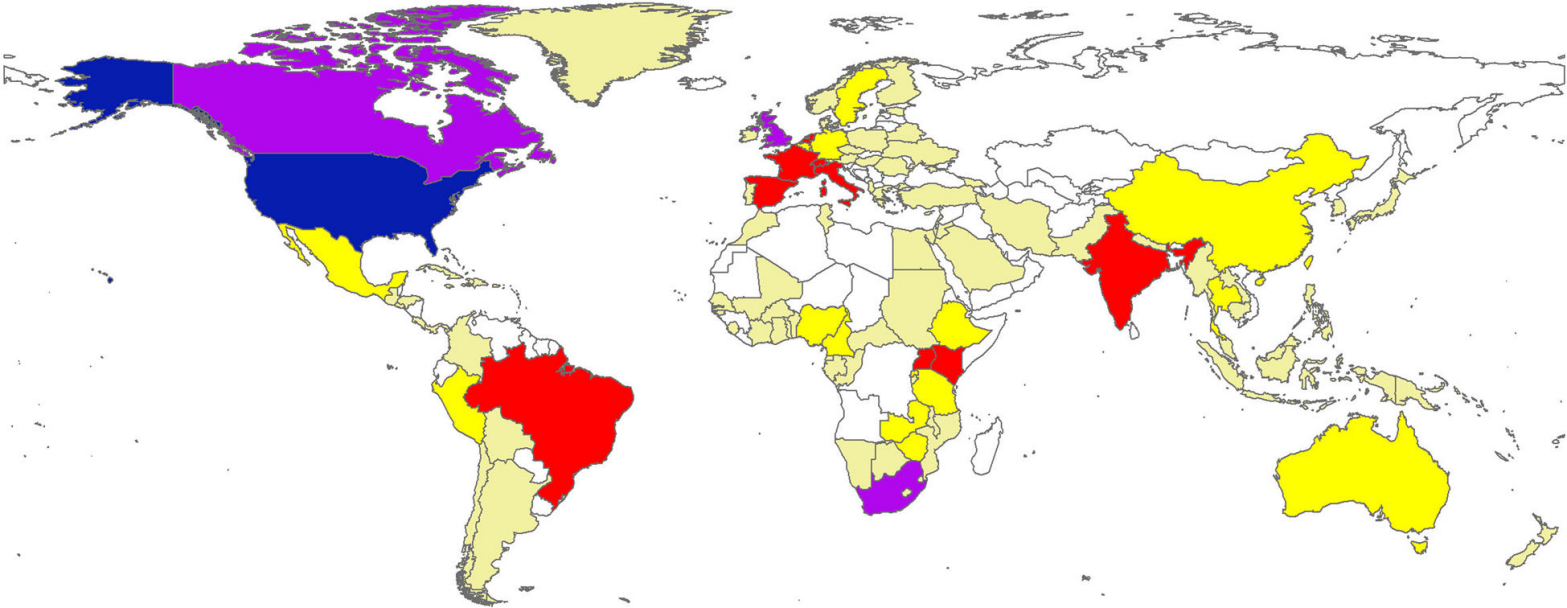
Geographical distribution of publications in HIV/AIDS – related medication adherence. The map was created using ArcMap program. The retrieved data were first exported from excel file into ArcMap and then layered over an empty world map available through the ArcMap program. The colors of various world region were selected by the program itself

Analysis of the retrieved documents indicated that authors in the region of Americas made the largest contribution (1931; 63.9%), followed by those in the European region (723; 23.9%), the region of Africa (497; 16.5%), the region of South East Asia (119; 3.9%), the Western Pacific region (106; 3.5%), and the Eastern Mediterranean region (11; 0.4%). Figure 4 shows the contribution of each WHO region to the retrieved documents as well as the number of publications per one million PLWH in each WHO region. Figure 4 showed that the region of Americas (567.9) had the highest research productivity stratified by number of PLWH (per one million) followed by the European region (314.3), Western Pacific Region (70.7), Eastern Mediterranean region (31.4), South East Asia (34.0), and Africa (19.3). Researchers from 102 countries contributed to retrieved literature. Figure 5 is a geographical mapping of retrieved documents.

### Most active countries

The list of these top ten active countries was shown in Table 4. The USA ranked first in research output (57.4%; *n* = 1733). Two countries in the top ten active list were from African region; one from Latin America, one from South East Asia, and the remaining were from Northern America and Western Europe. No significant correlation was found between the number of publications and the prevalence rate of HIV/AIDS. When the research output was standardized by calculating the percentage of publications on medication adherence relative to overall HIV/AIDS research activity in HIV/AIDS field, Uganda ranked first followed by Brazil and South Africa in.

**Table 4:** Top ten active countries in HIV/AIDS – related medication adherence

SCR^a^	Country	Number of publications *N* = 3021	%	Number of publications in HIV/AIDS field; *N* = 232,000	% of medication adherence publications from overall HIV/AIDS research	prevalence (%) of HIV among adults (15–49 years)
1st	United States	1733	57.4	91,600	1.9	0.6
2nd	South Africa	236	7.8	10,138	2.3	18.92
3rd	United Kingdom	199	6.6	18,915	1.1	0.33
4th	Canada	177	5.9	8362	2.1	0.3
5th	Spain	130	4.3	8334	1.6	0.42
6th	France	118	3.9	13,131	0.9	0.4
7th	Brazil	115	3.8	4974	2.3	0.55
8th	Uganda	102	3.4	2742	3.7	6.8
9th	Italy	80	2.6	9678	0.8	0.28
9th	Switzerland	80	2.6	5037	1.6	0.26

SCR, Standard competition ranking;

^a^ Equal countries have the same ranking number, and then a gap is left in the ranking numbers

### Most active institutions

Table 5 shows the top ten active institutions/organizations in the field of medication adherence among PLWH. All top ten active institutions/organizations were based in the USA. *Harvard University* (8.4%; *n* = 254) was the most active institution followed by *University of California, San Francisco* (7.4%; *n* = 223), *and Johns Hopkins University* (7.1%; *n* = 214). The top ten active institutions/organizations included seven academic institutions and three research/clinical organizations.

**Table 5:** Top ten active institutions/organizations in HIV/AIDS – related medication adherence

SCR^a^	Institution/organization	Number of publications N = 2031	%
1st	Harvard University	254	8.4
2nd	University of California, San Francisco	223	7.4
3rd	Johns Hopkins University	214	7.1
4th	Massachusetts General Hospital	148	4.9
5th	VA Medical Centers	124	4.1
6th	University of California, Los Angeles	111	3.7
7th	University of Washington, Seattle	102	3.4
8th	The University of North Carolina at Chapel Hill	95	3.1
9th	Centers for Disease Control and Prevention	91	3.0
10th	Columbia University in the City of New York	87	2.9

SCR, Standard competition ranking;

^a^ Equal institutions have the same ranking number, and then a gap is left in the ranking numbers

### International collaboration

**Fig. 6:**
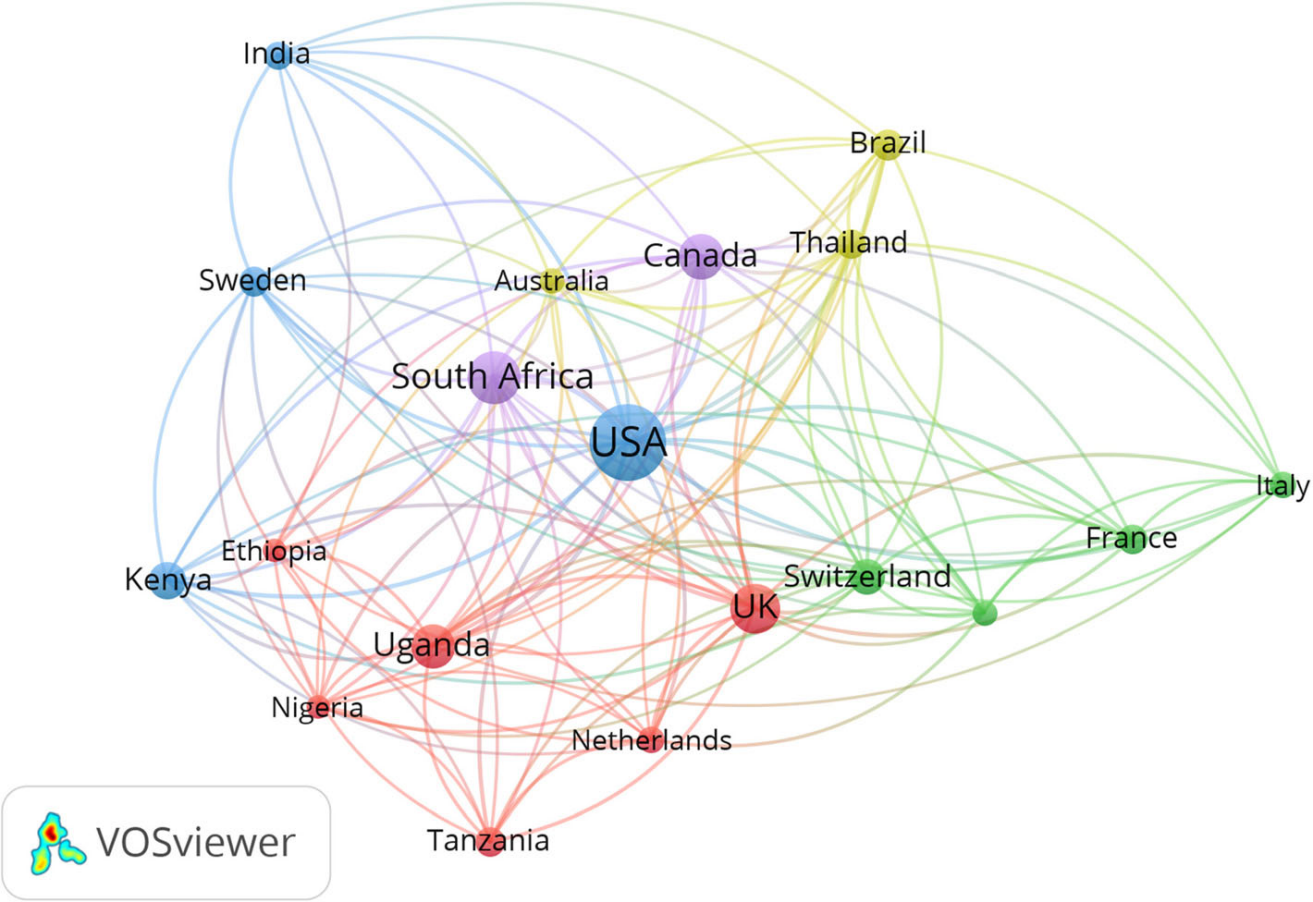
Network visualization map of international collaboration in countries with minimum productivity of 30 publications

International collaboration in the field of HIV/AIDS-related medication adherence research was visualized for countries with a minimum productivity of 40 publications (Fig. 6). The map shows that the USA occupies the center with many connecting line indicative of a large number of collaborating countries with the USA in this field. The relative strength of collaboration, measured by the thickness of the connecting lines between countries, was highest for USA – South Africa, USA – Uganda, a USA – Canada, USA – UK, and the USA – Kenya.

### Most preferred journals

In total, 634 different journals participated in publishing the retrieved documents; 39 of them were in the field of HIV/AIDS. The top ten active journals included eight journals in the field of HIV/AIDS, one in the field of infectious diseases, and one journal with multidisciplinary scope (Table 6). The top productive journal in this field was *AIDS Care* (8.0%; *n* = 241) followed by *AIDS and Behavior* journal (7.3%; *n* = 220). Six journals in the top active list were published from the USA, three from the UK, and one from the Netherlands.

**Table 6:** Top ten active journals in HIV/AIDS – related medication adherence

SCR^a^	*Journal Name*	Number of publications on medication adherence (%) N = 3021	%	Number of publications on HIV/AIDS in general (%) N = 232,000	%	Country
1st	AIDS Care	241	8.0	2691	9.0	UK
2nd	AIDS And Behavior	220	7.3	2239	9.8	Netherlands
3rd	Journal Of Acquired Immune Deficiency Syndromes	212	7.0	5403	3.9	USA
4th	AIDS Patient Care And STDs	173	5.7	1805	9.6	USA
5th	AIDS	104	3.4	8648	1.2	USA
6th	Plos One	95	3.1	3897	2.4	USA
7th	Journal Of The Association Of Nurses In AIDS Care	66	2.2	879	7.5	USA
8th	International Journal Of STD And AIDS	44	1.5	2162	2.0	USA
9th	Clinical Infectious Diseases	43	1.4	2955	1.5	UK
10th	HIV Clinical Trials	31	1.0	572	5.4	UK

SCR, Standard competition ranking;

^a^ Equal journalss have the same ranking number, and then a gap is left in the ranking numbers

### Authorship analysis

The average number of authors per document was 4.4. Table 7 is a list of top ten active authors in the field along with their affiliation as it appeared in Scopus. Professor Bangsberg, D.R. was the most prolific author (3.1%; *n* = 92) followed by Professor Safren, S.A. (1.7%; *n* = 49).

**Table 7:** Top ten active researchers (authors) in HIV/AIDS – related medication adherence

SCR^a^	Author name	Frequency	% (*N* = 3201)	Affiliation as shown in Scopus
1st	Bangsberg, D.R.	92	3.1	Massachusetts General Hospital, Division of Infectious Diseases, Boston, United States
2nd	Safren, S.A.	49	1.7	University of Miami, Coral Gables, United States
3rd	Montaner, J.S.G.	43	1.4	British Columbia Centre for Excellence in HIV-AIDS, Vancouver, Canada
3rd	Chesney, M.A.	43	1.4	UCSF School of Medicine, Osher Center for Integrative Medicine, San Francisco, United States
3rd	Gross, R.	43	1.4	University of Pennsylvania, Center for Clinical Epidemiology and Biostatistics, Philadelphia, United States
6th	Spire, B.	41	1.3	Sciences Economiques and Sociales de la Santé and Traitement de l’Information Médicale, Marseille, France
6th	Kalichman, S.C.	41	1.3	University of Connecticut, Department of Psychology, Storrs, United States
8th	Wilson, I.B.	35	1.2	Brown University, Department of Health and Human Services, Providence, United States
9th	Simoni, J.M.	33	1.1	University of Washington, Seattle, Department of Psychology, Seattle, United States
9th	Haberer, J.E.	33	1.1	Massachusetts General Hospital, Boston, United States
9th	Nachega, J.B.	33	1.1	Universiteit Stellenbosch, Department of Medicine and Centre for Infectious Diseases, Stellenbosch, South Africa
9th	Amico, K.R.	33	1.1	University of Michigan School of Public Health, Ann Arbor, United States

SCR, Standard competition ranking;

^a^ Equal authors have the same ranking number, and then a gap is left in the ranking numbers

### Highly cited articles

Highly cited articles on HIV/AIDS-related medication adherence were identified. The top cited articles for the period from 1992 to 2007 were shown in Additional file 2. The top cited article for the specified period received a total of 2410 citations, an average of 134 citations per year since the time of publication [53]. This study concluded that adherence to protease inhibitor therapy of 95% or greater gave an optimum virologic outcome in PLWH. The top cited articles for the period from 2008 to 2017 were also shown in Additional file 2**.** The top cited article received 599 citations, an average of 75 citations per year since the time of publication [54].

### Role of HIV-related stigma

**Fig. 7:**
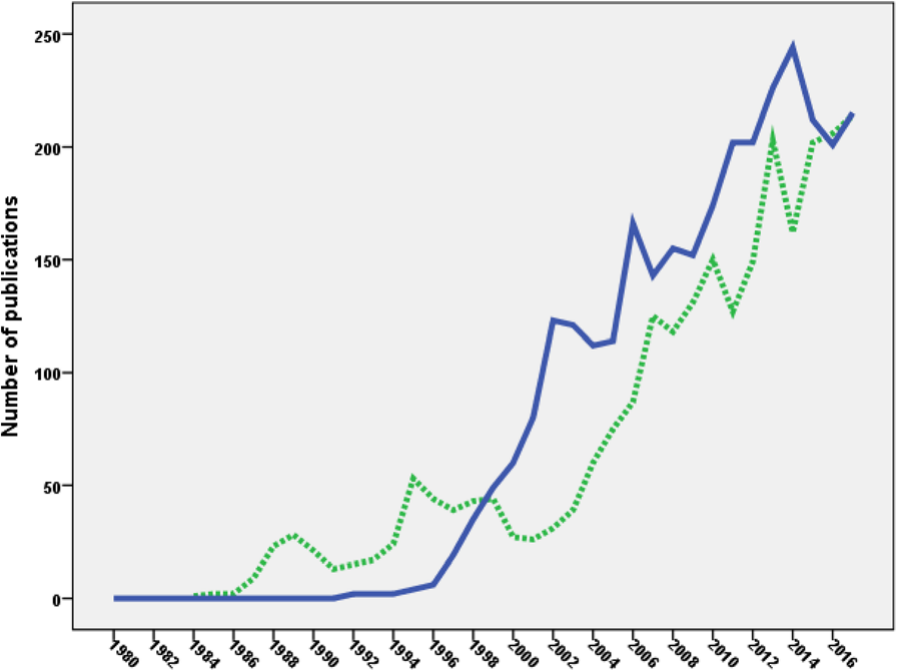
Comparative growth of publications on HIV/AIDS – related medication adherence (blue line) and HIV/AIDS – related stigma (green line)

Stigma research activity in HIV/AIDS showed parallel growth to that of medication adherence with a correlation coefficient of 0.912 and a *p*-value of < 0.01. Figure 7 shows the growth of publications in both HIV/AIDS-related stigma and HIV/AIDS-related medication adherence. Research activity on HIV/AIDS-related stigma started earlier than that on medication adherence. However, the number of publications on medication adherence was higher than that on AIDS-related stigma research. The total number of publications on HIV/AIDS-related stigma was 2510; approximately 83.1% of that on HIV/AIDS – related medication adherence research output.

### Role of mobile technology in medication adherence

In total, 149 (4.9%) documents about the role of mobile and phone technology in adherence to AIDS therapy were retrieved. These documents were published during the period from 2003 to 2017. The documents received 3643 citations, an average of 24.4 citations per document. The highest citation was achieved by an article published in *The Lancet* and the article discussed the role of short mobile messages in improving adherence to AIDS therapy in Kenya [54]. This study indicated a significant increase of adherence rate and a significant reduction in viral load in patients receiving phone messages as reminders of medication adherence. The second highly cited article in the role of mobile technology in adherence to AIDS therapy was also published on a group of patients in Sub-Saharan Africa and concluded that SMS reminders increased adherence in resource-limited settings [55].

## Discussion

### Growth of publications

This study aimed to analyze literature on HIV/AIDS-related medication adherence and present the results in bibliometric maps and tables. The results of this study showed a noticeable growth in this field relative to the overall HIV/AIDS research output indicative of the importance of this topic in the global fight against HIV/AIDS particularly in countries with limited resources or those with high social stigma against HIV infected people. The growth of publications in HIV/IDS-related medication adherence could be attributed to several factors. First, the natural increase in global research and publications on HIV/AIDS in general, secondary to international calls to decrease the global health burden of HIV/AIDS [56]. However, it should be emphasized that the results presented in this study indicated that the growth of publication on medication adherence showed relatively faster upward growth than that on HIV/AIDS in general. Second, the shift in HIV/AIDS disease paradigm from an acute untreatable infectious disease to a chronic illness that requires a high degree of adherence [57–59]. Third, the international aids and support to African countries with high prevalence of HIV/AIDS which created a momentum of international collaboration between developed countries and many African countries in research pertaining to HIV/AIDS including medication adherence research [60, 61]. The finding that Uganda and South Africa ranked among the top ten active countries at the global level indicated the role of international collaboration in fighting HIV/AIDS in Africa. Fourth, the introduction and advancement of mobile technology which provided a new frontier for medication adherence research in the field of HIV/AIDS [62, 63]. The current study showed that medication adherence research pertaining to HIV/AIDS started in the early 1990s while reports on HIV/AIDS started in the early 1980s, exhibiting a one-decade gap. This gap is most probably due to the advancement in HIV/AIDS therapeutic approaches, which resulted in HIV/AIDS being envisioned as a chronic illness that needs continuous adherence to medications.

### Geographical distribution of publications

HIV/AIDS is a global public health problem, with the problem in Africa considered to pose the greatest challenge, given a variety of barriers including poor access to medications, unsafe sex behaviors, stigma, and poverty. As a result, many African countries have significant numbers of PLWH [64, 65]. The contribution of African countries to medication adherence research was evident but inadequate given the health barriers and social challenges that PLWH might face in Africa. The contribution of certain countries such as South Africa and Uganda was quantitatively among the top active countries. However, when this research productivity was stratified with numbers of PLWH, the productivity was low. Relatively low research productivity from Africa and other world regions could be attributed to the limited number of clinicians and researchers in the field of infectious diseases in general and in the field of AIDS in particular. Furthermore, the lack of financial resources and academic funding needed to support publication fees in high-quality journals could be another potential reason for the limited contribution of African countries to global research output in this field. Researchers in Africa and other world regions are keen to publish and contribute to the advancement of health situation but the financial obstacles and academic responsibilities act as major barriers. Furthermore, regulations regarding academic tenure and promotion in scientific institutions in Africa and other world regions do not pressure researchers and clinicians to be engaged in research activity. One potential mechanism to encourage and increase research output from Africa and other world regions is through research networking and research collaboration with researchers in developed countries. Such collaboration can help researchers in Africa in manuscript writing and submission in addition to the potential of financial funding for publishing in high-quality journals. Actually, the findings of the current study indicated that the contribution of African countries to medication adherence research was made through international collaboration with developed countries such as USA, UK, and Canada.

### Most frequent author keywords

Results indicated that keywords such as stigma, depression, and drug abuse were most frequently encountered author keywords in HIV/AIDS-related medication adherence literature. Stigma has been reported, particularly in low and middle – income countries, to be a real and important barrier to medication adherence [66–71]. The findings that research activity in HIV/AIDS-related stigma was parallel to that of AIDS-related medication adherence is indicative of inter-relationship between stigma and adherence in PLWH [3, 15, 72–75]. The keyword “depression” showed up as a frequent author keyword possibly due to the role of depression in medication non-adherence. Patients with HIV/AIDS have impaired psychological health that could negatively affect their adherence to medications [76]. Substance abuse also showed up as a frequent author keyword possibly due to its role in medication non-adherence and mortality among users [77].

### Citation analysis

The finding that the *h*-index was 136 indicates that a large number of citations and a large number of readers showed interest in this field. For example, the *h*-index for publication on antimicrobial resistance of uropathogens [78], multidrug resistance tuberculosis [38], and anti-malarial drug resistance [79] was lower than that reported in the current study. Furthermore, the *h*-index obtained in this study was also higher than that reported for health topics unrelated to infectious diseases [37, 79, 80]. One potential explanation for the high *h*-index relative to other non-infectious diseases is the fact that non-adherence in HIV/AIDS patients might have more deleterious and acute effects than that for other chronic diseases such as diabetes. For example, medication non-adherence in diabetes could be replaced with diet restriction and exercise, which is not the case in HIV/AIDS. Furthermore, non-adherence in patients with HIV/AIDS might lead to a serious resistance problem to anti-viral drugs, which is not the case with anti-diabetic medications [81–86]. Medication adherence to anti-HIV medications is of extreme importance given that more than 95% adherence to HIV medications is required to minimize and suppress the viral load and control the disease, however this is not the case in chronic diseases such as diabetes mellitus where medium adherence might still be adequate in controlling the chronic disease [87–90].

### Most active journals, institutions, and authors

The most active journals in adherence to AIDS therapy were those in the specific field of AIDS. There are more than 70 journals in the specific field of AIDS/HIV indexed in Scopus. These journals created a scientific forum for researchers and clinicians to discuss hot topics in the field of AIDS. The involvement of these journals in publishing research in AIDS-related medication adherence is an indication of the high importance of this topic to the scientific community. However, most of these journals were published in Northern America and Europe, which might explain the large contribution of researchers from developed countries to publications appearing in these journals. Most institutions in the active list were also from high-income countries in Americas and Europe. It seems that academic institutions in the USA receive good funding for research pertaining to AIDS/HIV in general and to aspects pertaining to issues with serious impact on public health in particular. The presence of large numbers of PLWH in the USA is another potential reason for the high involvement of US researchers and institutions in AIDS-related medication adherence.

### Limitations and future direction

Medication adherence in PLWH is a global health issue and publication in this field is growing. It was the goal of this study to retrieve all relevant literature. However, not all journals, particularly those issued in Africa, are indexed in Scopus. Therefore, it is possible that some publications were missed. Furthermore, the title search gives accurate data but might cause some loss of publications in which the keywords were mentioned in abstract and not in the title. In this study, quality of publications was measure by citation analysis and *h*-index, which is not an ideal method to measure quality because self-citations might influence the values of citations and give a false impression of high impact. Despite these limitations, this study is the first to discuss literature on adherence to AIDS therapy in a bibliometric methodology. The findings of the current study should direct the efforts of researchers, human right activists, clinicians, and health policymakers to encourage research that point out barriers to medication adherence in world regions with a high burden of infected people. Furthermore, international health agencies need to direct and allocate funds and financial support to research in which technology could be used to overcome barriers to adherence. Social and human right activists need to speak out on the role of stigma and discrimination in medication adherence and such should implement social policies to minimize stigma and, therefore, enhance adherence. Clinicians in their practice settings should use mobile technology as a method of following up and communication with PLWH. This is extremely important in world regions where stigma and decimation as a real barrier to medication adherence. Clinicians should pressure the pharmaceutical industry and international health organization to provide AIDS therapy with minimum cost to low resourced regions. The Pharmaceutical industry could also help in improving adherence by manufacturing combination products with a minimum frequency of dosing and minimum possible cost. Nurses could also play a key role in improving adherence. Nurses in AIDS clinics should provide people with HIV full information regarding the importance of medication adherence in AIDS and the potential hazards of non-adherence to prescribed therapy. The fight against AIDS is a global and collective effort and people from the entire clinical spectrum, including clinical researchers, need to play a positive role in this regard.

## Conclusion

The results of this study showed (1) an increase in the number of publications with time and that this increase was parallel to that of AIDS-related stigma literature; (2) the region of Africa had the least research productivity in HIV/AIDS-related medication adherence when productivity was adjusted by the number of PLWH; (3) depression and stigma were one of the most frequent author keywords in HIV/AIDS-related medication adherence literature; finally (4) the *h*-index of retrieved literature was relatively high indicative of the importance of the topic to researchers and clinicians. Research on medication adherence in PLWH is a key component for the plan to minimize the global health burden of HIV/AIDS particularly in the presence of effective anti-HIV therapies that were proven to increase survival of PLWH. International research collaboration needs to be strengthened, particularly with countries where HIV/AIDS burden is high and national resources are scarce.

## Additional files

Additional file 1: Research strategy with keywords used and implemented to retrieve literature in HIV/AIDS – related medication adherence (DOC 32 kb)

Additional file 2: Top 10 cited documents on HIV/AIDS-medication adherence (DOCX 38 kb)

## Abbreviations

HIV AIDS: Human Immunodeficiency Virus Acquired Immunodeficiency Syndrome
PLWH: People living with HIV
WHO: World Health Organization
