# Supplementary material for: Global research output on HIV/AIDS–related medication adherence from 1980 to 2017
Source: BMC Health Serv Res. 2018 Oct 10;18:765. doi: 10.1186/s12913-018-3568-x (PMC6180611; doi:10.1186/s12913-018-3568-x)
Supplement: Supplementary file 1 — Research strategy with keywords used and implemented to retrieve literature in HIV/AIDS – related medication adherence (DOC 32 kb) [file 12913_2018_3568_MOESM1_ESM.doc]

**Screening**

**Included**

**Eligibility**

**Identification**

TITLE ( "medication adherence"  OR  "drug compliance"  OR  *adheren*  OR  "drug discontinuation"  OR  "drug persistence"  OR  "medication compliance"  OR  "patient* compliance"  OR  "patient* adherence"  OR  "prescription refill"  OR  "medication refill"  OR  "treatment refusal"  OR  adher*  OR  non-adher*  OR  "patient cooperat*"  OR  dropout*  OR  noncomplian* )

**N = 45,709**

Number of records after combining the two search queries
**N = 3,263**

Records screened and false positive results were excluded

**N = 3,062**

Exclusion :

NOT  TITLE ( bacteria  OR  atrial  OR  cardiac  OR  arterial  OR  dialysis  OR  lipid  OR  *hypertens*  OR  heart  OR  diet*  OR  mother  OR  parent  OR  family  OR  guideline  OR  appointment  OR  "compliance aids"  OR  "adherence aids"  OR  "Aids to drug compliance"  OR  "graphic aids"  OR  "visual aids"  OR  screening  OR  antibody  OR  macrophages  OR  cell  OR  dermatology  OR  candida  OR  apha  OR  keratino*  OR  leukocyte )

Eligible article for analysis

**N = 2,833**

articles excluded, with reasons

1. Published in 2017
2. Not journal articles

Studies included in bobliometric analysis

**N = 2,833**

( TITLE ( "human* immunodeficiency virus"  OR  "Acquired Immunodeficiency Virus"  OR  "Acquired Immunodeficiency Syndrome"  OR  "HIV Infect*"  OR  hiv  OR  "protease inhibitor*"  OR  "antiretrovir*"  OR  "anti-retrovir*"  OR  azt  OR  zidovudine  OR  haart  OR  "reverse transcriptase"  OR  "highly active" )  OR  ( TITLE ( aids )  AND  TITLE-ABS ( hiv ) )  OR  TITLE-ABS ( hiv  AND  aids ) )  AND  TITLE-ABS-KEY ( hiv  OR  aids  OR  infection  OR  virus  OR  viral )

**N = 273,174**
