# Supplementary material for: Global research output on HIV/AIDS–related medication adherence from 1980 to 2017
Source: BMC Health Serv Res. 2018 Oct 10;18:765. doi: 10.1186/s12913-018-3568-x (PMC6180611; doi:10.1186/s12913-018-3568-x)
Supplement: Supplementary file 2 — Top 10 cited documents on HIV/AIDS-medication adherence (DOCX 38 kb) [file 12913_2018_3568_MOESM2_ESM.docx]

**Global research output on HIV/AIDS–related medication adherence from 1980 to 2017**

**Waleed M. Sweileh**

**Additional file 2**

**Top cited articles in HIV/AIDS-related medication adherence**

Top ten cited articles in HIV/AIDS – related medication adherence for the period from 1992 – 2007 [[1-10](#_ENREF_1)]

| **Title** | **year** | **Name of the journal** | **Number of citations** | **Time elapsed since publication** | **Number of citations per year** |
| --- | --- | --- | --- | --- | --- |
| Adherence to protease inhibitor therapy and outcomes in patients with HIV infection | 2000 | *Annals of Internal Medicine* | 2410 | 18 | 133.9 |
| Self-reported adherence to antiretroviral medications among participants in HIV clinical trials: The AACTG Adherence Instruments | 2000 | *AIDS Care - Psychological and Socio-Medical Aspects of AIDS/HIV* | 992 | 18 | 55.1 |
| Adherence to protease inhibitors, HIV-1 viral load, and development of drug resistance in an indigent population | 2000 | *AIDS* | 794 | 18 | 44.1 |
| Non-adherence to highly active antiretroviral therapy predicts progression to AIDS | 2001 | *AIDS* | 629 | 17 | 37.0 |
| Adherence to antiretroviral therapy in sub-Saharan Africa and North America: A meta-analysis | 2006 | *Journal of the American Medical Association* | 608 | 14 | 43.4 |
| A comparison study of multiple measures of adherence to HIV protease inhibitors | 2001 | *Annals of Internal Medicine* | 578 | 17 | 34.0 |
| Patterns, correlates, and barriers to medication adherence among persons prescribed new treatments for HIV disease | 2000 | *Health Psychology* | 532 | 18 | 29.6 |
| Sociodemographic and psychological variables influencing adherence to antiretroviral therapy | 1999 | *AIDS* | 499 | 19 | 26.3 |
| Adherence to HAART: A systematic review of developed and developing nation patient-reported barriers and facilitators | 2006 | *PLoS Medicine* | 472 | 14 | 33.7 |
| The value of patient-reported adherence to antiretroviral therapy in predicting virologic and immunologic response | 1999 | *AIDS* | 448 | 21 | 21.3 |

Top ten cited articles in HIV/AIDS – related medication adherence for the period from 2011 – 2017 [[11-20](#_ENREF_11)]

| **Title** | **year** | **Name of the journal** | **Number of citations** | **Time elapsed since publication** | **Number of citations per year** |
| --- | --- | --- | --- | --- | --- |
| Effects of a mobile phone short message service on antiretroviral treatment adherence in Kenya (WelTel Kenya1): A randomised trial | 2010 | *The Lancet* | 599 | 8 | 74.9 |
| Mobile phone technologies improve adherence to antiretroviral treatment in a resource-limited setting: A randomized controlled trial of text message reminders | 2011 | *AIDS* | 441 | 7 | 63.0 |
| Depression and HIV/AIDS treatment nonadherence: A review and meta-analysis | 2011 | *Journal of Acquired Immune Deficiency Syndromes* | 272 | 7 | 38.9 |
| Interventions to improve adherence to self-administered medications for chronic diseases in the United States: A systematic review | 2012 | *Annals of Internal Medicine* | 252 | 6 | 42.0 |
| Alcohol use and antiretroviral adherence: Review and meta-analysis | 2009 | *Journal of Acquired Immune Deficiency Syndromes* | 230 | 9 | 25.6 |
| Explaining adherence success in sub-Saharan Africa: An ethnographic study | 2009 | *PLoS Medicine* | 221 | 9 | 24.6 |
| Mobile phone text messaging for promoting adherence to antiretroviral therapy in patients with HIV infection | 2012 | *Cochrane Database of Systematic Reviews* | 217 | 6 | 36.2 |
| Impact of HIV-related stigma on treatment adherence: systematic review and meta-synthesis. | 2013 | *Journal of the International AIDS Society* | 205 | 5 | 41.0 |
| A Randomized Controlled Trial of Cognitive Behavioral Therapy for Adherence and Depression (CBT-AD) in HIV-Infected Individuals | 2009 | *Health Psychology* | 200 | 9 | 22.2 |
| The combined effect of modern highly active antiretroviral therapy regimens and adherence on mortality over time | 2009 | *Journal of Acquired Immune Deficiency Syndromes* | 192 | 9 | 21.3 |

**References**

1. Bangsberg DR, Hecht FM, Charlebois ED, Zolopa AR, Holodniy M, Sheiner L, Bamberger JD, Chesney MA, Moss A: **Adherence to protease inhibitors, HIV-1 viral load, and development of drug resistance in an indigent population**. *AIDS* 2000, **14**(4):357-366.

2. Bangsberg DR, Perry S, Charlebois ED, Clark RA, Roberston M, Zolopa AR, Moss A: **Non-adherence to highly active antiretroviral therapy predicts progression to AIDS**. *AIDS* 2001, **15**(9):1181-1183.

3. Gordillo V, Del Amo J, Soriano V, González-Lahoz J: **Sociodemographic and psychological variables influencing adherence to antiretroviral therapy**. *AIDS* 1999, **13**(13):1763-1769.

4. Haubrich RH, Little SJ, Currier JS, Forthal DN, Kemper CA, Beall GN, Johnson D, Dubé MP, Hwang JY, McCutchan JA: **The value of patient-reported adherence to antiretroviral therapy in predicting virologic and immunologic response**. *AIDS* 1999, **13**(9):1099-1107.

5. Chesney MA, Ickovics JR, Chambers DB, Gifford AL, Neidig J, Zwickl B, Wu AW: **Self-reported adherence to antiretroviral medications among participants in HIV clinical trials: The AACTG Adherence Instruments**. *AIDS Care - Psychological and Socio-Medical Aspects of AIDS/HIV* 2000, **12**(3):255-266.

6. Liu H, Golin CE, Miller LG, Hays RD, Beck CK, Sanandaji S, Christian J, Maldonado T, Duran D, Kaplan AH *et al*: **A comparison study of multiple measures of adherence to HIV protease inhibitors**. *Annals of Internal Medicine* 2001, **134**(10):968-977.

7. Paterson DL, Swindells S, Mohr J, Brester M, Vergis EN, Squier C, Wagener MM, Singh N, Hudson B: **Adherence to protease inhibitor therapy and outcomes in patients with HIV infection**. *Annals of Internal Medicine* 2000, **133**(1):21-30.

8. Catz SL, Kelly JA, Bogart LM, Benotsch EG, McAuliffe TL: **Patterns, correlates, and barriers to medication adherence among persons prescribed new treatments for HIV disease**. *Health Psychology* 2000, **19**(2):124-133.

9. Mills EJ, Nachega JB, Buchan I, Orbinski J, Attaran A, Singh S, Rachlis B, Wu P, Cooper C, Thabane L *et al*: **Adherence to antiretroviral therapy in sub-Saharan Africa and North America: A meta-analysis**. *Journal of the American Medical Association* 2006, **296**(6):679-690.

10. Mills EJ, Nachega JB, Bangsberg DR, Singh S, Rachlis B, Wu P, Wilson K, Buchan I, Gill CJ, Cooper C: **Adherence to HAART: A systematic review of developed and developing nation patient-reported barriers and facilitators**. *PLoS Medicine* 2006, **3**(11):2039-2064.

11. Pop-Eleches C, Thirumurthy H, Habyarimana JP, Zivin JG, Goldstein MP, De Walque D, MacKeen L, Haberer J, Kimaiyo S, Sidle J *et al*: **Mobile phone technologies improve adherence to antiretroviral treatment in a resource-limited setting: A randomized controlled trial of text message reminders**. *AIDS* 2011, **25**(6):825-834.

12. Viswanathan M, Golin CE, Jones CD, Ashok M, Blalock SJ, Wines RCM, Coker-Schwimmer EJL, Rosen DL, Sista P, Lohr KN: **Interventions to improve adherence to self-administered medications for chronic diseases in the United States: A systematic review**. *Annals of Internal Medicine* 2012, **157**(11):785-795.

13. Horvath T, Azman H, Kennedy GE, Rutherford GW: **Mobile phone text messaging for promoting adherence to antiretroviral therapy in patients with HIV infection**. *Cochrane Database of Systematic Reviews* 2012, **2017**(12).

14. Safren SA, O'Cleirigh C, Tan JY, Raminani SR, Reilly LC, Otto MW, Mayer KH: **A Randomized Controlled Trial of Cognitive Behavioral Therapy for Adherence and Depression (CBT-AD) in HIV-Infected Individuals**. *Health Psychology* 2009, **28**(1):1-10.

15. Gonzalez JS, Batchelder AW, Psaros C, Safren SA: **Depression and HIV/AIDS treatment nonadherence: A review and meta-analysis**. *Journal of Acquired Immune Deficiency Syndromes* 2011, **58**(2):181-187.

16. Hendershot CS, Stoner SA, Pantalone DW, Simoni JM: **Alcohol use and antiretroviral adherence: Review and meta-analysis**. *Journal of Acquired Immune Deficiency Syndromes* 2009, **52**(2):180-202.

17. Lima VD, Harrigan R, Bangsberg DR, Hogg RS, Gross R, Yip B, Montaner JSG: **The combined effect of modern highly active antiretroviral therapy regimens and adherence on mortality over time**. *Journal of Acquired Immune Deficiency Syndromes* 2009, **50**(5):529-536.

18. Katz IT, Ryu AE, Onuegbu AG, Psaros C, Weiser SD, Bangsberg DR, Tsai AC: **Impact of HIV-related stigma on treatment adherence: systematic review and meta-synthesis**. *Journal of the International AIDS Society* 2013, **16**(3 Suppl 2).

19. Lester RT, Ritvo P, Mills EJ, Kariri A, Karanja S, Chung MH, Jack W, Habyarimana J, Sadatsafavi M, Najafzadeh M *et al*: **Effects of a mobile phone short message service on antiretroviral treatment adherence in Kenya (WelTel Kenya1): A randomised trial**. *The Lancet* 2010, **376**(9755):1838-1845.

20. Ware NC, Idoko J, Kaaya S, Biraro IA, Wyatt MA, Agbaji O, Chalamilla G, Bangsberg DR: **Explaining adherence success in sub-Saharan Africa: An ethnographic study**. *PLoS Medicine* 2009, **6**(1):0039-0047.
